# A Cristae-Like Microcompartment in *Desulfobacterota*

**DOI:** 10.1128/mbio.01613-22

**Published:** 2022-11-02

**Authors:** Shawn Erin McGlynn, Guy Perkins, Min Sub Sim, Mason Mackey, Thomas J. Deerinck, Andrea Thor, Sebastien Phan, Daniel Ballard, Mark H. Ellisman, Victoria J. Orphan

**Affiliations:** a Earth-Life Science Institute, Tokyo Institute of Technology, Meguro, Tokyo, Japan; b Center for Sustainable Resource Science, RIKEN, Hirosawa, Wako, Saitama, Japan; c Blue Marble Space Institute of Science, Seattle, Washington, USA; d National Center for Microscopy and Imaging Research (NCMIR), Center for Research in Biological Systems (CRBS), University of California, San Diego (UCSD), School of Medicine, La Jolla, California, USA; e Department of Neurosciences, University of California, San Diego (UCSD), La Jolla, California, USA; f School of Earth and Environmental Sciences, Seoul National University, South Korea; g Division of Geological and Planetary Sciences, California Institute of Technology, Pasadena, California, USA; University of California, Berkeley

**Keywords:** anaerobic oxidation of methane, sulfate reduction, *Desulfobacterota*, electron microscope tomography, intracytoplasmic membrane, outer membrane vesicles, pera, pera junction, compartmentalization

## Abstract

Some *Alphaproteobacteria* contain intracytoplasmic membranes (ICMs) and proteins homologous to those responsible for the mitochondrial cristae, an observation which has given rise to the hypothesis that the *Alphaproteobacteria* endosymbiont had already evolved cristae-like structures and functions. However, our knowledge of microbial fine structure is still limited, leaving open the possibility of structurally homologous ICMs outside the *Alphaproteobacteria*. Here, we report on the detailed characterization of lamellar cristae-like ICMs in environmental sulfate-reducing *Desulfobacterota* that form syntrophic partnerships with anaerobic methane-oxidizing (ANME) archaea. These structures are junction-bound to the cytoplasmic membrane and resemble the form seen in the lamellar cristae of opisthokont mitochondria. Extending these observations, we also characterized similar structures in Desulfovibrio carbinolicus, a close relative of the magnetotactic *D. magneticus*, which does not contain magnetosomes. Despite a remarkable structural similarity, the key proteins involved in cristae formation have not yet been identified in *Desulfobacterota*, suggesting that an analogous, but not a homologous, protein organization system developed during the evolution of some members of *Desulfobacterota*.

## INTRODUCTION

Structurally and functionally diverse intracytoplasmic membranes (ICMs) are found in organisms across the tree of life (1–7). A common feature of ICMs is that they compartmentalize bioenergetic processes that are linked to the production of ATP or to electron transfer, with their increased surface area leading to increased ATP production or membrane bound electron transport chain components (8, 9). The principal morphotypes of ICMs are (i) flat, long lamellae arranged in stacks, often around the cell periphery (4, 9–15), (ii) discs or vesicles, both attached and unattached to the cytoplasmic membrane (3), (iii) sparse, irregularly shaped invaginations (mesosomes) (7), and (iv) dark magnetosomes that align the cell along a geomagnetic field (9, 16, 17). ICMs can change shape, depending on the growth condition or stressor, (e.g., ammonia oxidation recovery or methane starvation [15, 18]), and inner membrane invagination can also create an enlarged periplasmic space (19). Cristae can also change their shape during development, and different cell types within the same organism can exhibit different crista morphotypes (20).

When the ICM is attached to the cytoplasmic membrane, the connection occurs through tubular membranous connections called ICM junctions (4, 9, 10, 13, 14). It has been hypothesized that ICM junctions compartmentalize bioenergetics by restricting the diffusion of metabolites, by localizing proton gradients, and by localizing particular membrane proteins to the ICM (9). In *Alphaproteobacteria*, ICM-afforded compartmentalization is thought to be facilitated by the equivalent of the mitochondrial contact site and cristae organizing system (MICOS) (9, 21–23). MICOS subunits in mitochondria, all with the base name Mic, are anchored at the mouth of the crista junction and, together with the soluble Opa1 protein, they form a cap at the crista junction mouth that can be opened by certain stimuli, such as apoptotic signals (6). A stabilizing subunit, Mic60, is a core anchor in the membrane of the crista junction mouth that recruits other subunits that form the junctional scaffold (6). There is direct experimental evidence for the involvement of alphaproteobacterial-derived Mic60 in photosynthetic ICM development (23). Because ICMs have been observed without ICM junctions in the form of un-attached “stacks” of membranes, (11), ICM junctions may not be always essential to the maintenance of ICMs, unlike the crucial role the crista junction plays in the formation and function of crista (22). This difference makes it important to identify whether or not cells with ICMs contain junctions.

The observation of ICMs in *Alphaproteobacteria* and the identification of proteins involved in their formation, some with homology to those in opisthokont mitochondria, led to the hypothesis that *Alphaproteobacteria* ICMs are the progenitor of the mitochondrial cristae (9, 20, 21, 23). Outside the *Alphaproteobacteria*, though, as discussed above, ICMs are common in several bacterial lineages and are affiliated with specific metabolic functions rather than taxonomic bacterial groups (i.e., ammonia and nitrite oxidation, methane oxidation, photosynthesis). In these other groups, direct attachment to the cytoplasmic membrane, as seen in the *Alphaproteobacteria* and opisthokont mitochondria, has become clearer. For example, in the methane-oxidizing gammaproteobacteria *M. alcaliphilum*, fluorescence recovery after photobleaching suggested membrane attachment (24). In other bacterial methanotrophs, membrane attachment could be seen under transmission electron microscopy (TEM) (10). In addition to these above-mentioned taxonomic and metabolic groups, ICMs have also been observed in *Desulfobacterota*, including Desulfovibrio carbinolicus (25), Desulfobacter postgatei (26), Desulfovibrio magneticus RS-1 (27), and *Candidatus* Magnetoglobus multicellularis (28). The aforementioned organisms are proposed as *Desulfobacterota* (29), whereas the taxonomy of *Candidatus* Magnetoglobus multicellularis has not yet been resolved (30, 31). However, whether these ICMs occur with an ICM junction remains unknown. These observations are of interest in our understanding of the structural evolution of cellular bioenergetics, given the hypothesized structural line of descent connecting the ICMs of an ancient anaerobic anoxygenic photosynthetic bacterium to those of the mitochondrial cristae, as well as to magnetosome-containing bacteria, aerobic methanotrophs, anoxygenic photosynthetic organisms, and nitrifiers (9). If a homologous structure exists in the *Desulfobacterota*, this finding could push the earliest occurrence of ICMs deeper into the bacterial phylogenetic tree. Alternatively, structurally similar but phylogenetically nonhomologous genes that are involved in ICM junction formation could point to convergent structural evolution in *Desulfobacterota* and *Alphaproteobacteria*.

To gain structural information regarding *Desulfobacterota* ICMs from both cultured and environmental microorganisms, we used electron microscope tomography (EMT) to visualize membrane invaginations and junctions. These observations demonstrate that the membrane invaginations observed in *D. carbinolicus* and in uncultured sulfate-reducing bacterial partners of methanotrophic ANME-2 archaea (referred to as Seep SRB1a and Seep SRB1g) (24, 32, 33) are directly connected to the cytoplasmic membrane. This is remarkably similar to the connection of cristae to the inner boundary membrane. We also analyzed the prominent intracellular granules and outer membrane vesicles (OMVs) associated with these environmental, syntrophic, sulfate-reducing bacteria.

## RESULTS

### 3,3′-diaminobenzidine (DAB) staining of bacterial cytoplasmic intrusions.

**FIG 1:**
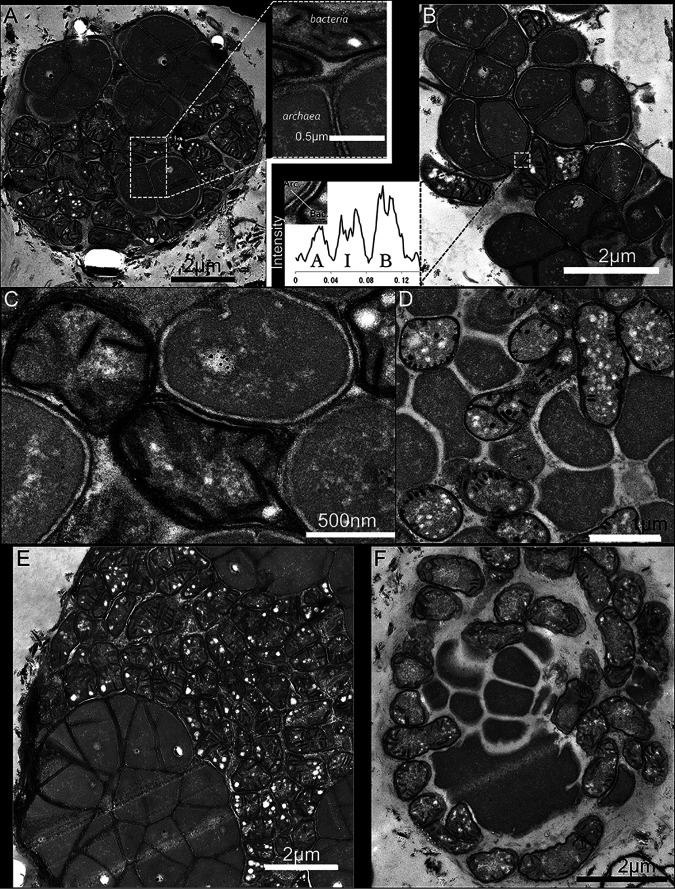
Application of DAB to seep derived microbial consortia to investigate the positioning of transition metal-based redox activity. (A) Panel and inset: ANME-SRB consortia showing the strong staining of bacterial ICMs by DAB. (B) ANME-SRB consortia showing the staining of bacterial ICMs with the inset showing (left to right) the stain intensity profile of the archaeal membrane (A), the intercellular location of the putative heme proteins that are involved in electron transfer (I), and the bacterial membranes (B). The *x* axis is measured in μm. Cells with morphological similarity to ANME-2b (which contain polyphosphate-like bodies) are stained by DAB on the cell exterior, indicating redox activity outside the cell (Panels A, B, C, and E). Other cells which experienced the same reaction conditions but do not appear to be ANME-2b (and are likely 2c) did not show staining between cells (Panels D and F). Panels A, B, and C are from the 5133 methane seep sample, and panels D, E, and F are from the 3730 methane seep sample.

In our initial observation of ANME-SRB consortia using TEM, we applied DAB to investigate the spatial localization of multiheme cytochrome proteins on the exterior of ANME cells (34). In addition to staining the exterior of the ANME cells, both the archaeal and bacterial cell membranes were stained, with the staining extending into the cytoplasm of the bacterial cells (Fig. 1). This staining is consistent with the presence of transition metal ions, which are capable of shuttling electrons from DAB to H_2_O_2_ in the assay.

### Electron microscope tomography (EMT) of environmental archaea-bacteria consortia.

EMT provided a high-resolution, three-dimensional (3D) visualization of the *Desulfobacterota* cells in this study; Fig. S1 provides low-magnification electron micrographs of the archaea-bacteria consortia. To indicate structural distinction and to not cause confusion by using the term, “crista-like”, which would give the erroneous impression that this class of ICMs has a similar function and protein complement to cristae, we propose to call this class of ICMs “pera,” meaning “pouch” in Latin. Note that pera is both the singular form and the plural form, so whether singular or plural is deduced from the context. This decision was made via analogy with “crista” meaning “crest” in Latin.

### Pera and pera junctions in syntrophic sulfate-reducing bacterial cells paired with ANME archaea derived from methane seeps.

**FIG 2:**
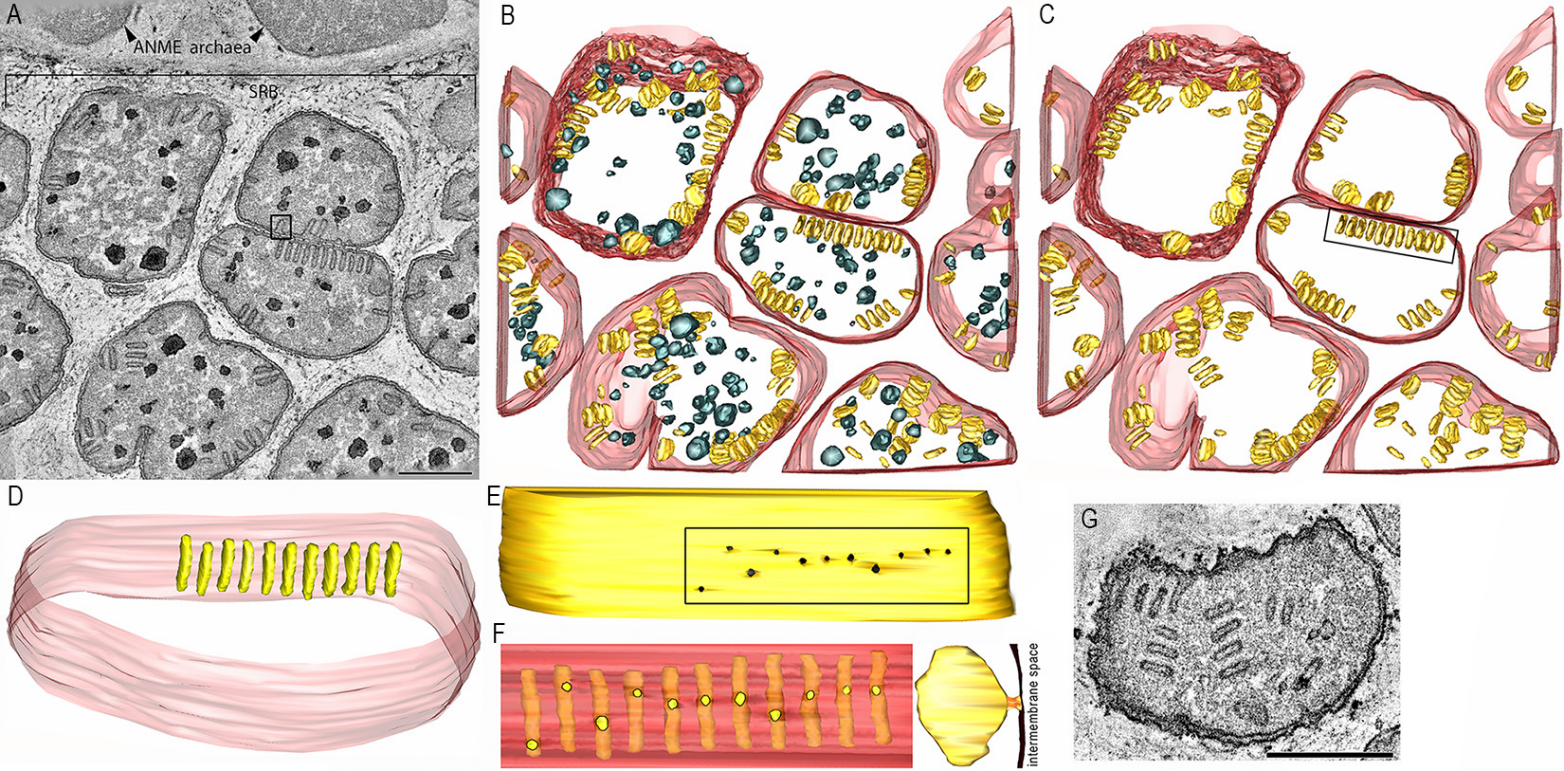
The T3B1 morphotype has short, stubby pera. (A) A 1.6 nm thick slice through the middle of a tomographic volume of a T3B1 ANME-SRB consortium. The ANME archaea were spatially separated from their partner SRB cells, as noted by the demarcating line. The SRB clustered in aggregates. Uniformly appearing pera are clearly seen by their dark membrane staining inside the profiles of 3 whole and 6 partially visualized SRB. Dark granules are prominent in the SRB. An example of a pera with a pera junction is boxed. Scale = 500 nm. (B) A “top” view of the surface-rendered volume shown in panel A after the segmentation of the cell (outer) membranes (transparent maroon color), dark granules (slate gray color), and pera (goldenrod color). (C) The pera are arranged mostly around the cell periphery and in rows. The boxed region of 11 pera is the most prominent example. (D) A side view of the pera in the boxed region, showing uniformity when viewed from the top and also from the side. (E) The cytoplasmic membrane, viewed from the outside, showing the 11 pera junctions of the pera in the boxed region. The pera junction openings (dark) are also strikingly uniform in size. The boxed region is shown in panel F. (F) In the left panel, with the cytoplasmic membrane made transparent, the placement of the pera junctions along the height of their respective pera is clear. Even though the pera junctions tend toward the middle of the pera, there is sufficient variation to conclude that the pera junction placement is not uniform. Rather, these junctions can appear anywhere along the pera height. The right panel shows the perpendicular view of one of the middle pera to show that a pera junction is a short, tube-like structure that connects the pera interior across the cytoplasmic membrane (translucent maroon) to the periplasmic space. This view emphasizes why pera, Latin for pouch, appropriately describes this structure. (G) Even though the preponderance of pera is arrayed along the periphery of the cell, there are examples of pera that appear to be in the middle of the cell. However, because the slice thickness only captures less than half of the cell, they may be closer to the “top” or “bottom” of the cell. Scale = 500 nm.

Based on previous studies of the same incubations in which archaea-bacteria pairs were analyzed (34–36), these bacteria are tentatively assigned in this study as *Desulfobacterota* Seep SRB1a and Seep SRB1g that form syntrophic partnerships with ANME-2 archaea. From the two sediment incubations analyzed, groupings could be made by comparing the structures of the pera. The T3B1 morphotype exhibited short, stubby pera with uniform appearance (Fig. 2A, C). The pera were arranged mostly around the cell periphery and in rows (Fig. 2C, D). Dark granules were prominent (Fig. 2A, B). The vast majority of the pera had a single pera junction that was a short, tube-like structure connecting the pera interior (lumen) across the cytoplasmic membrane to the periplasm (Fig. 2A, E, F). Like the pera, the pera junctions were also uniform in size (Fig. 2E, F). Even though the pera junctions tended toward the middle of the edge of the pera that was adjacent to the cytoplasmic membrane, there was sufficient variation to conclude that the placement of pera junctions was not uniform. Rather, they could appear anywhere along the pera base. Even though the preponderance of the pera were arrayed along the periphery of the cell, there were examples of pera in the middle of the cell. These few pera did not have a pera junction (Fig. 2G).

**FIG 3:**
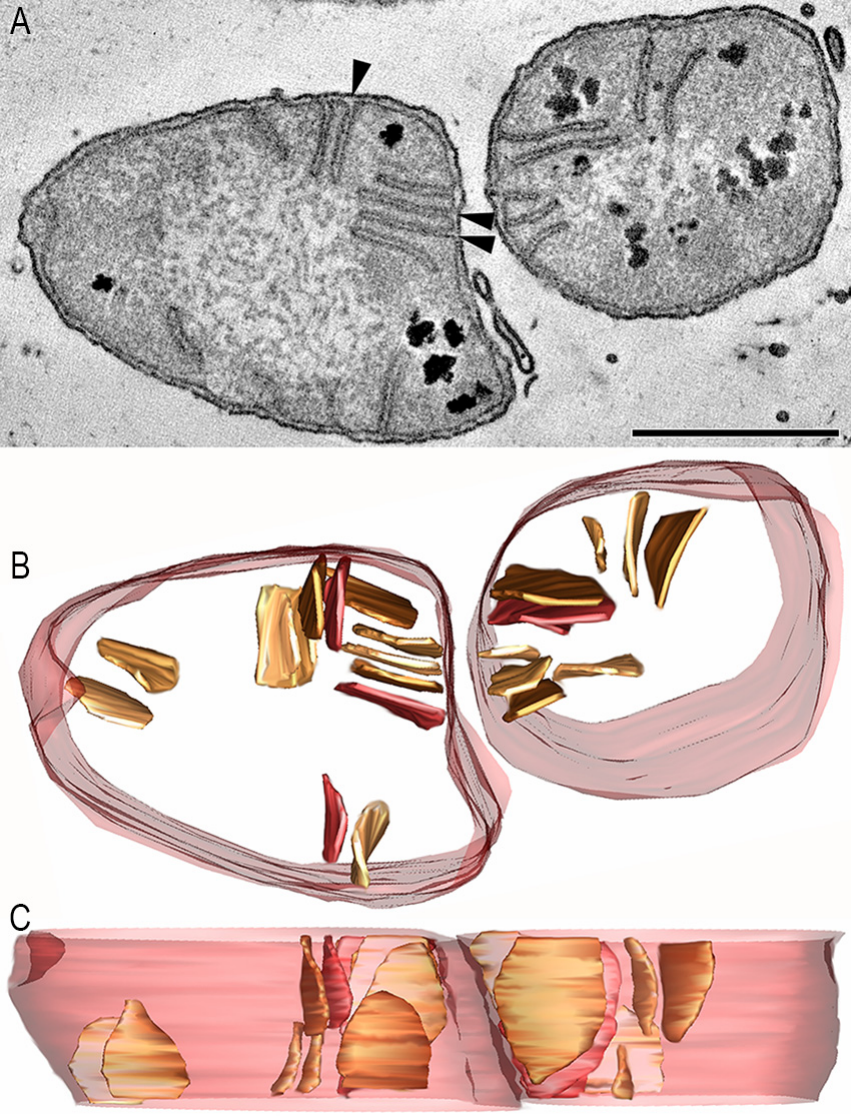
The T3B2 SRB morphotype has narrower, longer pera, compared with the T3B1 morphotype. (A) A 1.6 nm thick slice through the middle of a tomographic volume of an ANME-SRB consortium, showing 2 SRB cells. The pera lengths are noticeably less uniform than their T3B1 counterparts. Five open pera junctions are seen (arrowheads). The other pera junctions are closed, as seen by a cap across their entrance. As with the T3B1, the dark granules are prominent. Scale = 500 nm. (B) A “top” view of the surface-rendered volume after the segmentation of the cell (outer) membranes (transparent maroon) and pera (various shades of brown). Whereas the short, stubby pera found in the T3B1 cells were invariably straight, these pera are curvier. (C) Perpendicular view showing the difference in sizes of these pera.

**FIG 4:**
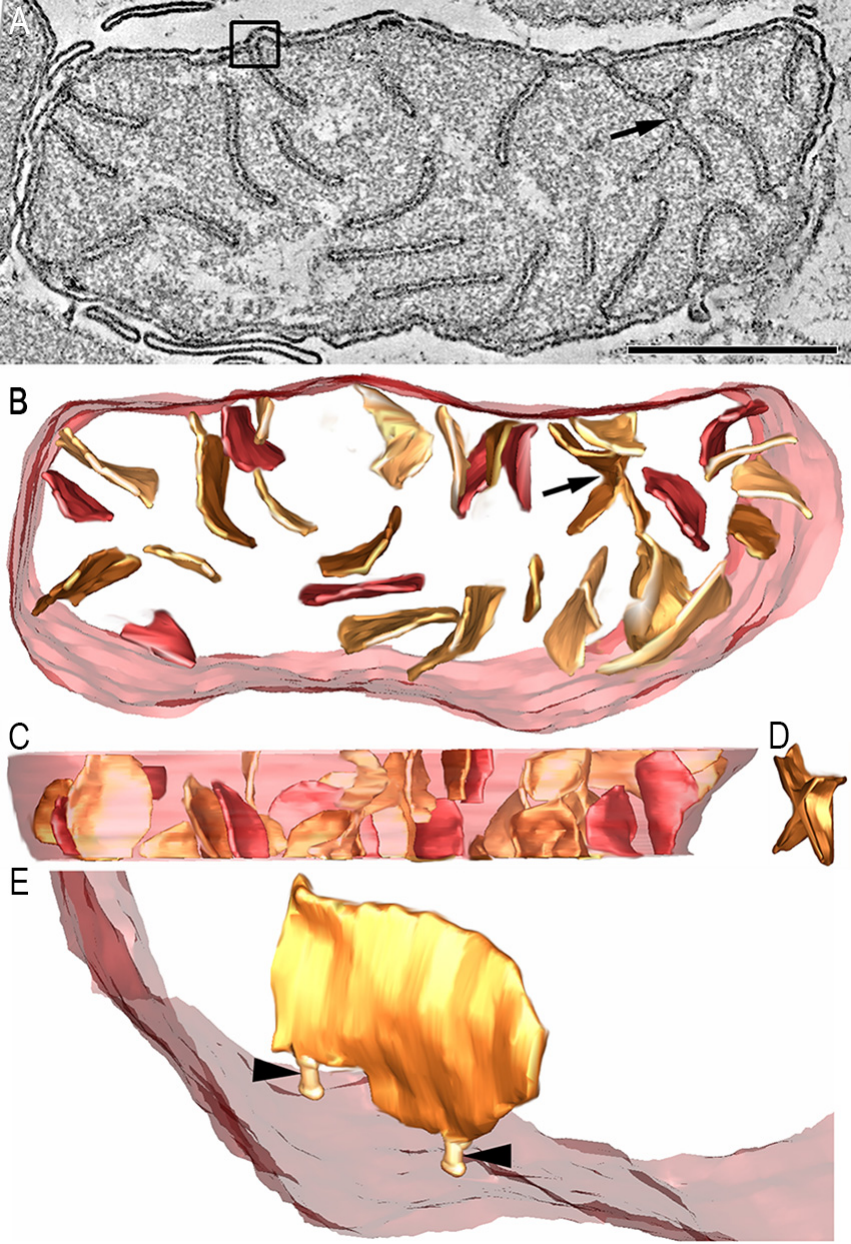
The T2B SRB morphotype has even longer and curvier pera, compared with the T3B2 morphotype, and it closely resembles the typical mitochondrial architecture. (A) A 1.6 nm thick slice through the middle of a tomographic volume of an ANME-SRB consortium, showing an SRB cell. No dark granule is seen. This cell could easily be mistaken for a mitochondrion because of the shape, size, and distribution of the pera, which resemble cristae. Although not common, there is even branching of the pera (arrow), reminiscent of branched cristae. Moreover, the pera junctions (example is boxed) look identical to crista junctions. Scale = 500 nm. (B) A “top” view of the surface-rendered volume after the segmentation of the cell membrane (transparent maroon) and pera (various shades of brown). These pera look like lamellar mitochondrial cristae. (C) Perpendicular view showing that these pera are larger than those in the T3B1 and T3B2 morphotypes. (D) An oblique view of the branched pera to better see that the branches are not connected by tubes but are rather joined throughout. (E) Oblique view of a pera (chocolate), emphasizing the pera junction (sandy brown) shape. This pera has 2 pera junctions (arrowhead), differing from the T3B1 pera, which have at the most only 1 pera junction each (Fig. 2F), and is more similar to mitochondrial lamellar cristae, which often have more than 1 crista junction.

A second morphotype from the same sediment push core, T3B2, had narrower, longer pera than those of the T3B1 morphotype (Fig. 3). The pera length was noticeably less uniform than the T3B1 pera length. Three open pera junctions were observed (Fig. 3A; arrowheads). The other pera junctions appeared to be closed, as evidenced by caps across their entrances. As observed with T3B1, dark granules were also prominent in the bacterial cells of this consortium. Whereas the T3B1 pera were invariably straight, these pera were curvier. Also, in contrast to the T3B1 SRB, there were fewer pera in arrays. The T2B samples came from an AOM consortium that was recovered from a different push core, and their pera were longer, larger (more surface area), and curvier than those of the T3B2 pera morphotype (Fig. 4).

### Electron microscope tomography (EMT) of sulfate-reducing bacteria Desulfovibrio carbinolicus.

**FIG 5:**
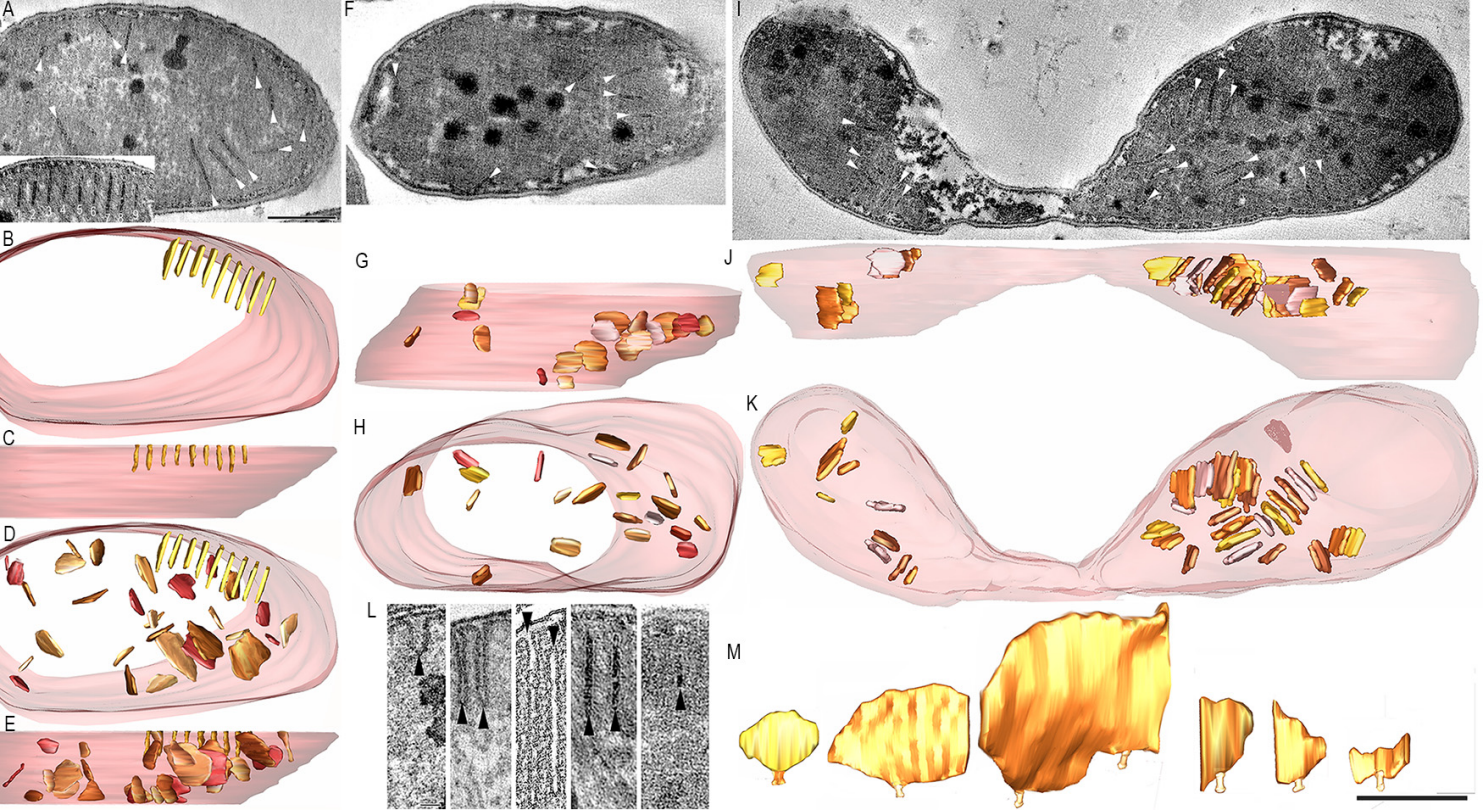
The DC morphotype has narrow, straight pera. (A) A 1.6 nm thick slice through the middle of a tomographic volume of a DC1 cell from a late exponential culture which had been transferred to grow on EtOH from MeOH. The contrast is reversed so that the pera membrane is light and the interior is dark. This reversal makes it harder to identify the pera, so each pera is marked by a white arrowhead. As with the T3B1 and T3B2 SRB, the dark granules are prominent. The inset shows another slice through the same volume that is closer to the section surface, showing pera arranged at the cell periphery and in a row, resembling the predominant arrangement of the T3B1 pera. These pera are numbered from 1 to 9 to help mark each. Scale = 250 nm. (B) A “top” view of the 9 surface-rendered pera shown in the inset after the segmentation of the cell membrane (transparent maroon) and pera (goldenrod). The regular array of pera was reminiscent of the T3B1 morphotype, except that the T3B1 pera were shorter and stubbier, and these pera were narrower and longer. (C) Perpendicular view showing that these pera are short. (D) A top view of all of the pera in this cell. There appear to be 2 classes of pera in the DC1 cells. One class (goldenrod) is T3B1-like and is arranged in an array but is narrower. The other class is comprised of pera of divergent orientation, but not random orientation, of different sizes (shades of brown).These pera are straight, unlike the curvier pera of the T2B morphotype. (E) Perpendicular view reinforcing the impression that the second class of pera is not randomly oriented but tends to be within 30 degrees of vertical. (F) A 1.6 nm thick slice through the middle of a tomographic volume of a DC2 cell from an early stationary culture which had been transferred to grow on EtOH from MeOH. Each pera is marked by a white arrowhead. The dark granules are also prominent with the DC2 cells. (G) A side view of all of the pera in this cell. (H) A perpendicular view, reinforcing the observation that many of the pera are positioned along the periphery of the cell. (I) A 1.6 nm thick slice through the middle of a tomographic volume of a DC3 cell grown in culture in the exponential-phase on EtOH from a culture grown on EtOH. Each pera is marked by a white arrowhead. As with the DC1 and the DC2 cells, the dark granules are also prominent with the DC3 cells. (J) A side view of all of the pera in this cell. (K) A perpendicular view, showing two interesting features. One, there tend to be separate aggregations of pera, with a few on the left to more than 10 on the right. Two, because we can track most of the pera completely through the volume, those pera in the middle of the cell cannot have pera junctions. (L) Side-by-side comparison of pera length from pera chosen near the median length for each type. Images were taken from slices of the volumes. From left to right: T3B1, T3B2, T2B, DC1, and DC3. An arrowhead points to each pera. DC2 (not shown) was similar to DC1. Scale = 50 nm. (M) Side-by-side comparison of the segmented pera with sizes chosen near the median for each type. Surface-rendered pera volumes are shown. Scale = 200 nm.

The cultured isolate Desulfovibrio carbinolicus (DC) also has pera. The pera morphotype of this sulfate-reducing bacterium was narrow, straight, and sometimes arranged in a row at the cell periphery (Fig. 5A). There appeared to be 2 classes of pera in the DC1 cells. One is morphologically similar to T3B1 and arranged in a row but is narrower and not as uniform (Fig. 5B, C). The other pera morphotype was made up of different sizes and had a divergent orientation, but it was not randomly oriented (Fig. 5D, E). Yet, these pera were straight, unlike the curvier pera of the T2B morphotype. With the DC1, 2, and 3 samples, there were pera in the cytoplasmic volume of the cell which were not connected to the cytoplasmic membrane and did not have pera junctions (Fig. 5D, H, K). As with the T3B1 and T3B2 SRB, dark granules were prominent in the DC cells (Fig. 5A, F, I). A side-by-side comparison of pera length is instructive, as it gives a graphic impression of how different the typical lengths are among the cultured and uncultured SRB groups included in our study (Fig. 5L). It is also useful to provide a side-by-side comparison of the pera sizes from the surface-rendered pera volumes. Here, the T2B pera dwarf the other pera imaged, with the pera in DC cells being on the small end of the scale, relative to the pera of both T2B and T3B2, but about the same size as the T3B1 pera (Fig. 5M).

**FIG 6:**
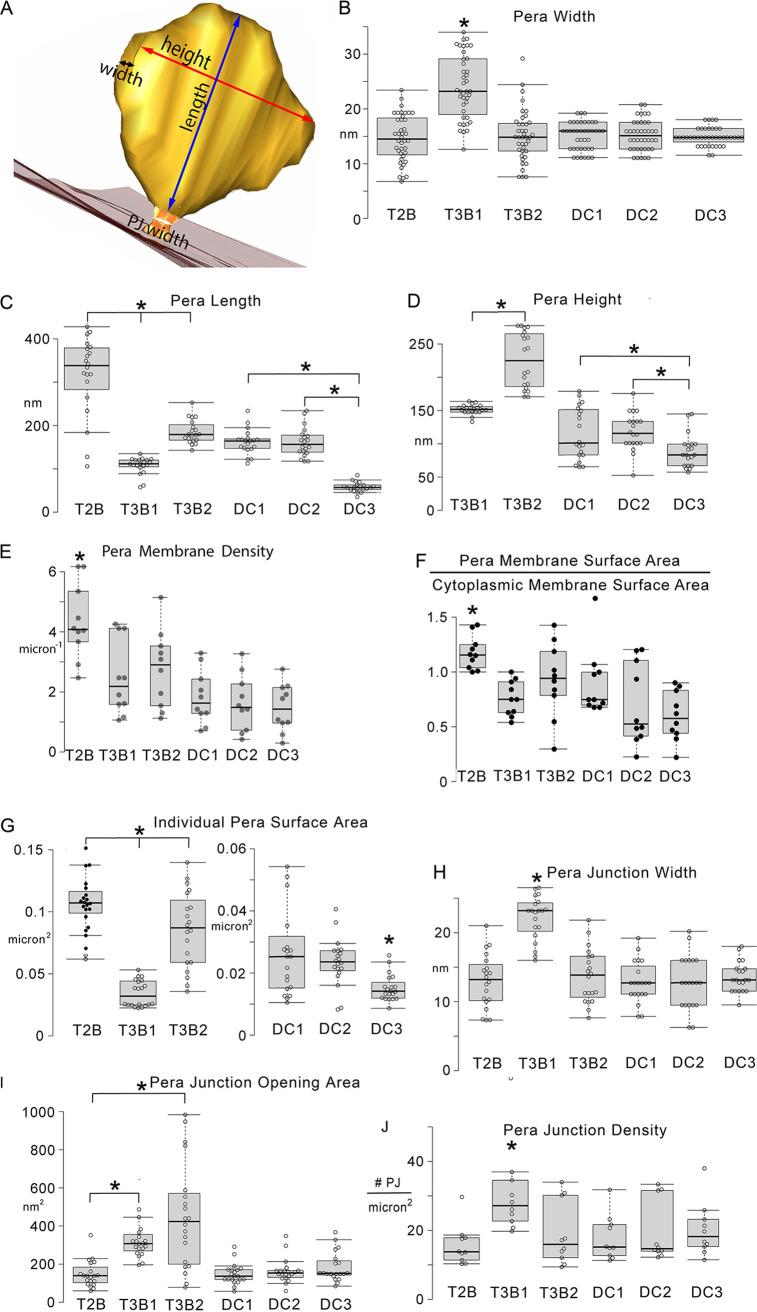
Measurements of pera and pera junction features. (A) Oblique view of a pera (chocolate) connected to the inner boundary membrane (translucent maroon), showing how the width, length, and height measurements were made. The black double arrow corresponds to the pera width. The blue double arrow corresponds to the pera length. The red double arrow corresponds to the pera height. The white double arrow corresponds to the pera junction (sandy brown) width. (B) The pera of the T3B1 morphotype have considerably greater maximum widths than do the pera of the T3B2 or T2B morphotypes, reenforcing the impression that these are stubbier pouches (*, *P* < 0.01; *n* = 40; from 10 cells each for T2B, T3B1, T3B2, DC1, DC2, and DC3).The DC1, DC2, and DC3 pera all have similar maximum widths that are no different from T3B2 or T2B. The maximum widths for 5 out of the 6 morphotypes are remarkably uniform. (C) The maximum pera lengths are strikingly different for T3B1, T3B2, and T2B, with the T3B1 being the shortest, reinforcing the impression that the T3B1 pera are short and stubby. Among the DC morphotypes, the DC3 pera are significantly shorter (*, *P* < 0.01; *n* = 20; 10 cells for each SRB type). (D) The maximum pera height was also different between the morphotypes, with the T3B1 being shorter than the T3B2 or T2B. Note that it was not possible to accurately measure the maximum height for the T2B pera because most extended beyond the section thickness of 300 nm used for the tomographic analyses. Clearly, though, this morphotype had the tallest pera (Fig. 5M). Among the DC samples, DC3 had pera shorter than those of DC1 or DC2 (*, *P* < 0.01; *n* = 20; 10 cells for each SRB type). (E) The pera membrane density is highest for the T2B morphotype (*, *P* < 0.01; *n* = 10 cells for each SRB type). (F) The pera membrane surface area divided by the cytoplasmic membrane surface area was greatest for the T2B morphotype, which was the only morphotype with a mean (and median) ratio that was greater than 1 (*, *P* < 0.01; *n* = 10 cells for each SRB type). (G) Individually, the T3B1 and DC3 pera had the least membrane surface area. Note that the *y* scale is different for the DC samples because their pera are generally smaller than those of T2B, T3B1, and T3B2. (*, *P* < 0.01; *n* = 20; 10 cells for each SRB type). (H) The pera junction width is greater for T3B1 than for the other morphotypes (*, *P* < 0.01; *n* = 20; 10 cells for each SRB type). (I) The Pera junction opening (cross-sectional) area at the pera junction entrance. The T3B1 and T3B2 cells have larger openings. This is the case for the T3B1 cells because they have larger circular openings and for the T3B2 because they have elongated (oblong) openings (*, *P* < 0.01; *n* = 20; 10 cells for each SRB type). (J) The pera junction density is greatest for the T3B1 pera, consistent with the observation of more pera, hence more pera junctions, per cellular volume in this morphotype, compared with the other morphotypes (*, *P* < 0.01; *n* = 10 cells for each SRB type).

Pera and pera junction structural features differ among cells and between samples, but they are consistent within a cell. Structural dimensions and densities were measured on EMT volumes (Fig. 6). Generally, the T2B, T3B1, and T3B2 morphotypes were compared with each other, and the DC1, 2, and 3 morphotypes were compared with each other, separately from the environmental morphotypes designated with “T”. The maximum widths for 5 out of the 6 morphotypes were remarkably uniform (Fig. 6B). Only the pera of the T3B1 morphotype had a considerably greater maximum width, reinforcing the impression that these are stubby pouches. The maximum pera lengths were considerably different for T3B1, T3B2, and T2B, with the T3B1 being the shortest, again in line with the impression that the T3B1 pera are short and stubby (Fig. 6C). Among the DC morphotypes, the DC3 pera were significantly shorter. The maximum pera lengths were considerably different for T3B1, T3B2, and T2B, with the T3B1 being the shortest, again consistent with T3B1 pera being short and stubby (Fig. 6C). The T2B maximum pera height could not be accurately measured, as many pera extended beyond the volume height, a feature reminiscent of lamellar cristae. Among the cultured DC samples, DC3, grown with ethanol and harvested at the early exponential phase, had pera which were shorter than those of the DC1 and DC2 cells innoculated from cells grown on methanol. The pera membrane density, defined as the total pera membrane surface area for a cell divided by the cell volume, for the T2B cells was higher than that measured for the T3B1 morphotype or the T3B2 morphotype (Fig. 6E). The DC morphotypes tended to have lower pera membrane densities than the environmental “T” morphotypes, but they were only statistically significantly lower than the T2B cells. The ratio of pera membrane surface area to cytoplasmic membrane surface area provides a measure of how much the pera membrane has been expanded, presumably to accommodate sulfate respiration or electron uptake, in the case of the syntrophic SRB, to satisfy the energy demands of a given cell (Fig. 6F). Only the T2B morphotype had more pera membrane than cytoplasmic membrane, on average. Individually, the T2B pera had more membrane surface area than did the T3B2 pera (Fig. 6G). Unsurprisingly, the T3B1 pera individually had the least membrane surface area, as they were short and stubby. The DC3 pera were not stubby, but they were short (Fig. 5M). Thus, they had less membrane surface area than did DC1 and DC2. The pera junction width for the stubbier T3B1 pera, measured between the points of the greatest membrane curvature at either side of the entrance to the pera junction, was also greater those of the other morphotypes (Fig. 6H). A comparison can be made with the crista junction width measurements from Table S1 from (20), using the lamellar cristae + tubular crista junction rows. From this meta-analysis, the median crista junction diameter was 28 nm with a standard deviation of 11 nm (*n* = 34 publications). The pera junctions were remarkably similar to crista junctions, with median values of 26 to 27 nm and the T3B1 morphotype representing the only one at the high end of the crista junction comparison at 37 nm. These values were calculated after adding the membrane widths (bilayer width from both sides of the junctional opening). The pera junction opening (cross-sectional) area at the pera junction entrance (Fig. 6I) reflects the circular nature of the openings for T3B1, T2B, DC1, DC2, and DC3. The T3B1 opening area was larger because the pera junction width (diameter) was greater (Fig. 6H). In contrast, the T3B2 cells had elongated (oblong) pera junction openings with a short axis of a similar width to the diameters of the circular per junction openings. The pera junction density, defined as the number of junctions per square micron of cell membrane surface area, was greatest for the T3B1 pera (Fig. 6J), reflecting that while the pera were significantly smaller (Fig. 6G), there were more pera, hence more pera junctions, per cellular volume in the T3B1 morphotype, compared with the other morphotypes.

10.1128/mbio.01613-22.4 TABLE S1 Bacterial homologs of known proteins involved in cristae formation. Download Table S1, DOCX file, 0.01 MB. Copyright © 2022 McGlynn et al. 2022 McGlynn et al. https://creativecommons.org/licenses/by/4.0/ This content is distributed under the terms of the Creative Commons Attribution 4.0 International license.

### Bacterial granules and membrane budding.

**FIG 7:**
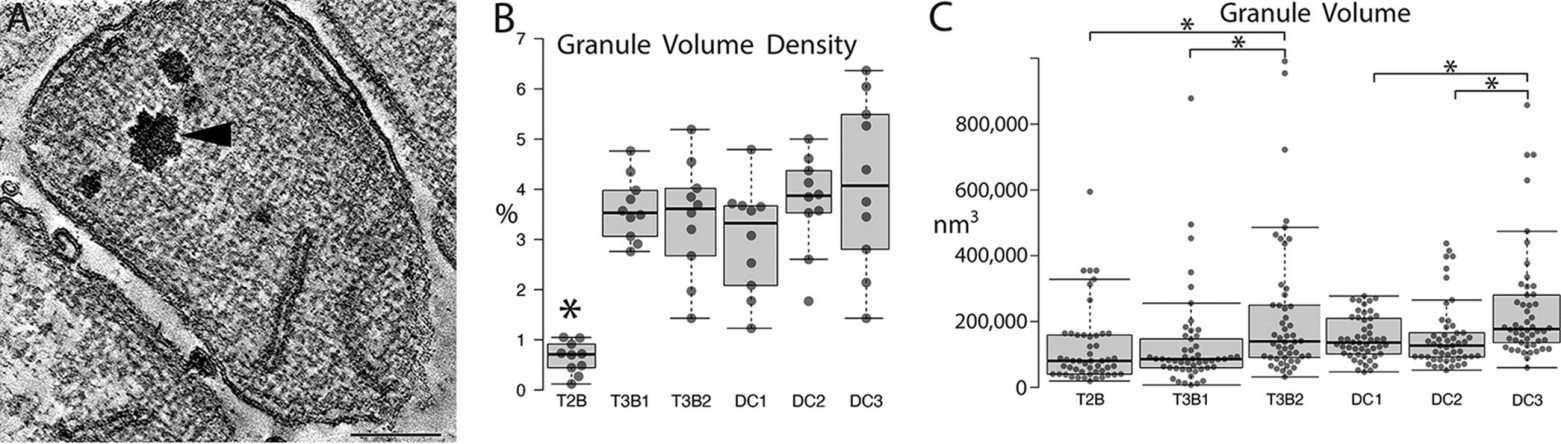
Dense granules, present in both methane seep and cultured bacteria, differ in volume, density, and size between the 6 classes of SRB. (A) An example of a T2B SRB containing a few large, dense granules that appear similar to granules prevalent in T3B1, T3B2, and DC1-3 SRB. An arrowhead points to a large granule. Scale = 200 nm. (B) The granule volume density is much lower in T2B SRB compared with T3B1, T3B2, and DC1-3 SRB (*P* < 0.01; *n* = 10 cells). (C) The granule volume is bigger for T3B2 SRB than for T2B or T3B1 SRB, and the DC3 granule volume is bigger than the volume of either DC1 or DC2 (*P* < 0.05; *n* = 50 granules).

A dominant structural feature present in both the methane seep syntrophic SRB partners of ANME archaea and cultured *D. carbinolicus* SRB was the occurrence of large, dense intracellular granules with irregular margins (Fig. 7). While we are unable to assign an elemental composition at this time, it is likely that they are glycogen granules, which are known to be a common carbon storage granule in *Desulfobacterota* (27, 37). Lead stains are known to bind glycogen (38), and here, intracellular material acquired contrast with the application of Sato’s lead stain (compare Fig. S2B and D). We are unaware of data showing the lead staining of either polyhydroxyalkanoates (PHA) or poly-B-hydroxybutyrate (PHB), so we tentatively identify these bodies as being comprised of carbohydrates or possibly polyphosphates. It can also be noted that we failed to obtain positive results when attempting staining with the Nile red stain, an indicator of PHA/PHB (38, 39). The most prominent examples of these bacterial inclusions occurred in cells that paired with archaea that were morphologically similar to ANME-2b (35). Bacteria paired with other ANME archaea contained a nonuniformly stained cytoplasm and contained fewer storage type inclusions (Fig. S2E and F). The granule volume density (i.e., the percentage of the cytoplasmic volume occupied by granules) was much lower in T2B SRB, compared with T3B1, T3B2, and the cultured DC1-3 SRB cells, which did not differ appreciably between the 5 types. In addition, the granule size (volume) was the largest for T3B2 and the ethanol-grown DC3 (Fig. 7C).

10.1128/mbio.01613-22.2 FIG S1 Low-magnification TEM images of the consortia used for the EM tomography in this study. Download FIG S1, TIF file, 2.7 MB. Copyright © 2022 McGlynn et al. 2022 McGlynn et al. https://creativecommons.org/licenses/by/4.0/ This content is distributed under the terms of the Creative Commons Attribution 4.0 International license.

10.1128/mbio.01613-22.3 FIG S2 TEM images of consortia from traditional uranyl acetate and OsO_4_ treatments as well as from the same preparation after Sato’s lead staining procedure. Bacteria associated with archaea with morphological similarity to ANME-2b contain intracellular granules which appear white (A and B). After the application of Sato’s lead stain, the granules appear to have been stained (C and D). Note that these images are from two different consortia. Panels E and F show bacteria pairing with archaea of a non-ANME-2b morphology. These contain some intracellular granules, but the granules lack the prominence of those shown above (see reference (6) for a description of the ANME-2b morphotype). The dashed white boxes in panels A, C, E, and F indicate the position zoomed images, which are at the right. The consortia in panels A–E were from the 3730 (T3B in this study) methane seep sample, and the consortium in panel F was from the 5133 microcosm (T2B in this study). Download FIG S2, TIF file, 2.5 MB. Copyright © 2022 McGlynn et al. 2022 McGlynn et al. https://creativecommons.org/licenses/by/4.0/ This content is distributed under the terms of the Creative Commons Attribution 4.0 International license.

**FIG 8:**
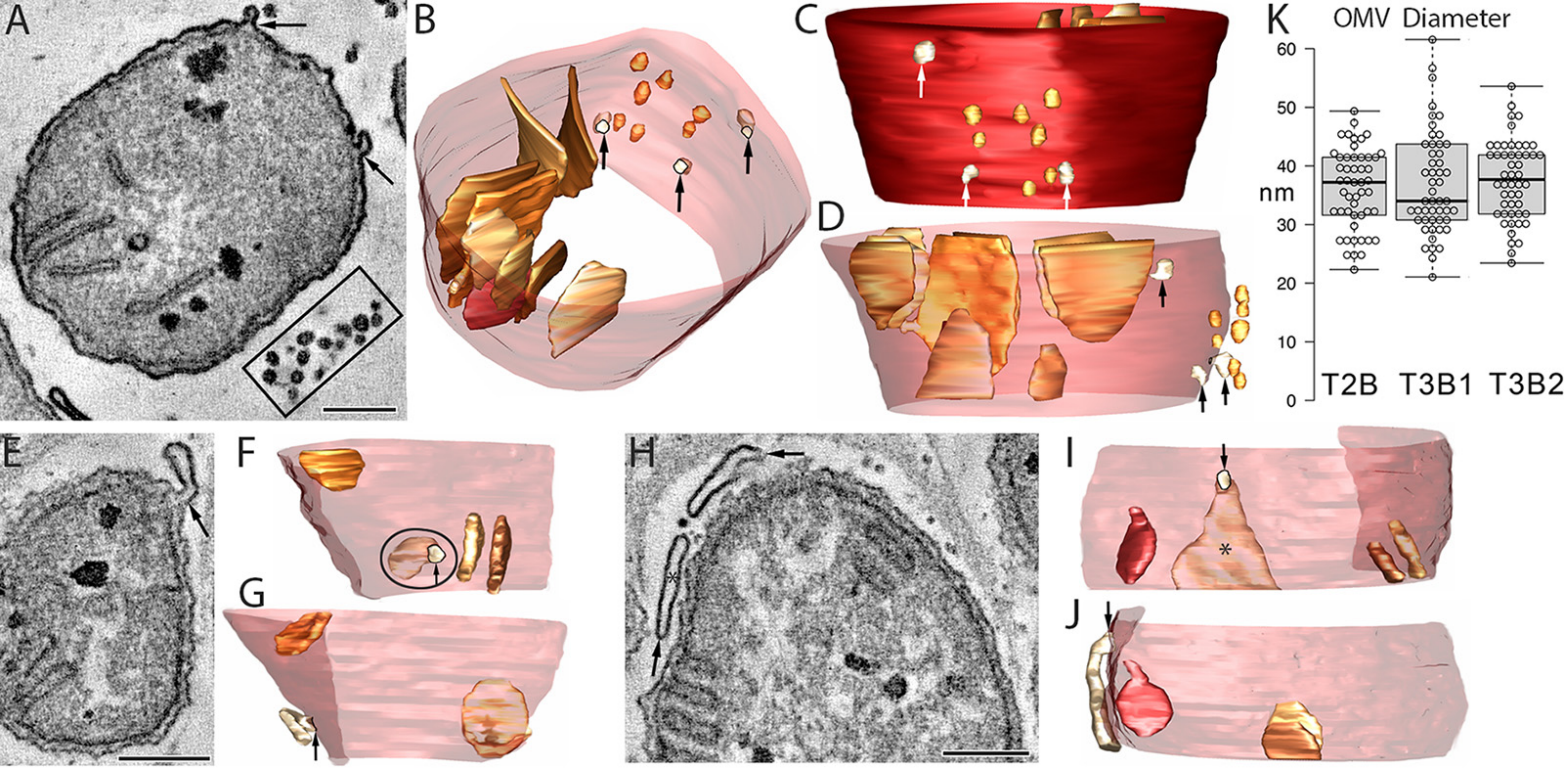
OMVs bud from the outer (cell) membrane of T2B, T3B1, and T3B2 SRB. (A) A T2B SRB showing 2 buds (arrows) and a nearby cluster of spherical OMVs (boxed). Scale = 200 nm. (B) A view from the top, angled to look through the cell membrane made translucent so as to see the nearby OMVs, three of which are still attached to the outer membrane (arrows). All of the pera are shown to demonstrate that they are positioned in a subvolume that is separated from the portion of the outer membrane where the OMV budding is observed. (C) A side view of the adjacent OMVs against the backdrop of the opaque outer membrane, providing another perspective of the distribution of the OMVs. (D) Another side view, giving a good perspective of how close these OMVs are to the outer membrane after budding off. The large pera typical of T2B SRB provide a structural landmark reference to the size of the OMVs. (E) Even though the vast majority of the OMVs are spherical, lamellar-type OMVs are also observed. An arrow points to a lamellar-type OMV budding from the cell membrane of a T3B2 SRB. Scale = 200 nm. (F) A side view, looking through the translucent outer membrane, partly cut away, at the lamellar OMV (circled), with the budding portion indicated by an arrow. The smallish pera are shown for perspective. (G) A rotated side view, showing how the lamellar OMV parallels the outer membrane. An arrow points to the attached portion of the OMV. (H) Large lamellar OMVs (arrows) are also observed adjacent to the SRB outer membranes. Part of a T3B1 SRB is shown. Scale = 200 nm. (I) The lamellar OMV marked with an asterisk in panels H and I is attached to the outer membrane with a tubular bud (arrow). A side view with the outer membrane partially cut away to show that this lamellar OMV is larger than the pera. 3 are shown. (J) A rotated side view shows how this lamellar OMV is closely apposed to the outer membrane. (K) Measurements of the diameter of the spherical-type OMVs, with examples shown in panel A. The diameters were relatively uniform and did not vary between T2B, T3B1, or T3B2 (*n* = 50).

Outer membrane vesicles (OMVs) that bud from the cell membrane of the T2B, T3B1, and T3B2 syntrophic SRB were commonly observed in the methane oxidizing consortia. Any OMVs budding from DC1–3 were likely lost during the pelleting process used for sample preparation. The most common type of OMV was the small, spherical type (Fig. 8A). Sometimes, the OMVs were clustered (Fig. 8A, boxed). The budding process was commonly observed, indicating active OMV creation within sediment-hosted consortia at the time of sample fixation (Fig. 8A–D). Usually, the pera, and more particularly, the pera junctions, were positioned in subvolumes that were distinct from the portion of the cell membrane where the OMV budding was observed (Fig. 8B). Even though the majority of the OMVs were spherical, lamellar-type OMVs were also common (Fig. 8E–G). Large lamellar OMVs were also observed adjacent to the SRB cell membranes, sometimes attached by membranous tubes to the cell membranes (Fig. 8H–J). The spherical-type OMVs displayed relatively uniform diameters that were, interestingly, about the same as the diameters of the tubular bud to lamellar OMV. There appeared to be no correlation between OMV type (spherical or lamellar) in relation to the type of SRB based on pera structure (short and stubby or long, thin, and curvy) (data not shown).

## DISCUSSION

A foundational theme in both bacterial and mitochondrial biology is that mechanisms have evolved to match inner membrane architecture to the needs of the cell. It has been hypothesized that ICMs and cristae are specialized microcompartments which optimize bioenergetics, that is, the greater the energy requirements, the more ICM/cristae surface area (40–42). However, there is still much to learn in our understanding of the structural and functional ICMs across biology; only a small sample of microbial diversity has been investigated in structural detail. By using high resolution 3D-EMT, we have explored the unique lamellar ICMs in the syntrophic SRB bacterial partners of ANME archaea in environmental methane-oxidizing consortia and in the sulfate-reducing bacterial isolate *D. carbinolicus*.

### Structure of *Desulfobacterota* pera.

The T2B pera SRB morphotype was remarkably similar to that of mammalian mitochondria. The cell shown in Fig. 4A could easily be mistaken for a mammalian mitochondrion because of the shape, size, and distribution of the pera, which closely resemble those of the lamellar cristae of opisthokont mitochondria (20). Although not common, branching of pera was observed (Fig. 4A, B, D), reminiscent of branched cristae. Moreover, the pera junctions looked identical to metazoan crista junctions. Some pera had multiple pera junctions (Fig. 4E), whereas others were limited to one pera junction each (Fig. 2F) and were more similar to mitochondrial lamellar cristae, which often have more than one crista junction in metazoans (20).

The T3B1 SRB represented a morphotype that least resembled opisthokont mitochondria, presenting with short and stubby pera that were remarkably uniform in size and frequently packed closely together in rows (Fig. 2). These pera were morphologically more similar to the discoid, or paddle-like, cristae of many discobans, such as the kinetoplastid Trypanosoma (43). The placement and size of the pera were not random within the cell.

While there are clear morphological differences observed among the pera of these SRB, the structures of the three classes of pera were not mutually exclusive. For example, the T2B SRB pera resembled the T3B2 pera, except that they were larger. There are also similarities with the smaller, curvier pera commonly observed in DC1–3. In comparison, the second subclass of pera described from DC1 had a strong resemblance with the short and stubby T3B1 pera, including lining up in a row, except that they were thinner (Fig. 5B). The pera membrane density in these sulfate-reducing bacteria (Fig. 6E) was 5 to 10 times lower than that typically observed for metazoan crista membrane density (44–47). This lower density can be attributed to the finding that either the pera did not extend far into the interior, as was typical for the T3B1 pera (Fig. 2C), or that the pera tended to cluster together, leaving portions of the tomogram volume pera-free, as was found with the T3B2 pera (Fig. 3). Even the long pera of T2B SRB tended not to extend beyond half of the cell width (Fig. 4). In mitochondria, ATP is generated in the cristae; however, the specific locations of the ATP synthase and the reducing equivalents in *Desulfobacterota*, whether in the pera or elsewhere in the cytoplasmic membrane, have not yet been resolved.

### Diversity of membrane invaginations in *Desulfobacterota*.

Other than the data presented here, there are a limited number of TEM bacterial examples that show apparent inner membrane invaginations that are morphologically similar to those reported here. Early work by Thauer described internal membranes from the TEM data of Desulfobacter postgatei, which grew through dissimilatory sulfate reduction with acetate as the electron donor (26). Nanninga and coworkers published TEM images of ICMs in D. carbinolicus (Desulfovibrio sp. strain EDK82, also studied here), in which ICMs were reported to occur when grown on either ethanol or lactate but not when grown with methanol oxidation coupled to sulfate reduction (25). Third is the observation of an invaginated membrane observed in Desulfovibrio magneticus, a close relative of *D. carbinolicus*, grown on pyruvate coupled with fumarate as the electron acceptor (27). With the exception of Desulfovibrio magneticus, it was not determined whether the ICMs in these other organisms were physically connected to the cytoplasmic membrane or were a physically independent intracellular feature. The TEM images of organisms interpreted to be sulfate-reducing bacteria in anaerobic methane-oxidizing microbial mats from the Black Sea Crimean shelf, related to the environmental *Desulfobacterota* in our study, have also been reported to contain ICMs (48, 49). These observations, together with others on *Desulfobacterota* members, including Desulfobacter postgatei (26), Desulfovibrio magneticus RS-1 (27), and *Candidatus* Magnetoglobus multicellularis (28), suggest that pera might be widespread in this group. Follow-up studies are needed to investigate ICMs in these organisms, as well as to understand the biochemical basis for these structures and their evolutionary history.

### Redox activity of invaginated membranes in syntrophic SRB paired with ANME archaea.

Diaminobenzidine (DAB) is oxidized in a H_2_O_2_-dependent process by various iron complexes, notably protein-bound heme groups (50–55). Used in conjunction with TEM, DAB is widely used with the application of peroxidase-linked antibodies as a means of localizing various molecules in the cell, where peroxidase catalyzed deposition of the DAB polymer followed by osmification allows for the subcellular localization of antigens (56). It is also known to form a precipitate upon reaction with the cytochrome c oxidase present in aerobic organisms’ respiratory chains (57). In the case of anaerobic methane-oxidizing ANME-SRB consortia, cytochrome c oxidase is not present, as these organisms do not utilize O_2_ as a terminal electron acceptor. In ANME and SRB cells, then, DAB reaction products should stem from endogenous transition metal complexes that are capable of electron transfer (redox) reactions. These are expected to exist within the respiratory chains of the organisms (in the cases of ANME and SRB, these complexes will be in the membrane), as well as, to a lesser extent, in the cytoplasm of the cell. As SRB house their respiratory chains within the cytoplasmic membrane, these invaginations could house respiratory enzymes, leading to an enhanced respiratory capacity due to an increased cell surface area and ability to pack a greater number of these enzymes into a given cell, analogous to the membrane-associated particulate methane monooxygenase in aerobic methanotrophs (58). While we have not yet obtained DAB staining data on the organisms listed in the above paragraph, we consider it likely that they will stain redox active, as the ANME-associated bacteria did.

### Evolutionary implications of a crista-analogous structure in *Desulfobacterota*.

It has been proposed that cristae evolved from *Alphaproteobacterial* ICMs (9, 20, 21, 23). However, *D. carbinolicus* and the presumed ANME-partner *Desulfobacterota* SRB studied here display pera that more closely resemble opisthokont cristae than *Alphaproteobacterial* ICMs. Further, because the tubulo-vesicular crista shape is much more phylogenetically widespread across eukaryotes (20), it is not likely that the lamellar crista type is ancestral to eukaryotes.

What type of protein is involved with pera junction formation? And, is there even an equivalent to the MICOS in the cells that we studied? Mic60 (mitofilin domain-containing) is phylogenetically restricted to the *Alphaproteobacteria* and mitochondria (23, 59, 60) and is without homologs of the key protein components involved in cristae formation in *Desulfobacterota* (i.e., Mic60, 10, 12, 19, 26, and other homologs) (Table S1). *Desulfobacterota* may have convergently evolved a structure similar to that found in opisthokont mitochondria. The proteins involved in the formation of pera are currently unknown, though it is fascinating that invaginated *Desulfobacterota* ICMs have been hypothesized to lead to the eukaryotic endomembrane system (61).

One piece of the puzzle of pera formation may involve the lipid cardiolipin. The presence of cardiolipin in both bacterial and mitochondrial membranes has been argued as evidence supporting the endosymbiotic origin of the mitochondrion (42, 62). Cardiolipin plays a crucial role in cristae formation and stabilization in metazoans, fungi, and plants (20, 63). The cardiolipin content is higher in ICMs, compared with the cell or cytoplasmic membranes (40, 64). Therefore, it is likely that cardiolipin plays a role in the formation of ICMs. Whether this formative role is a possibility with the pera subclass of ICMs remains to be determined, but it is worth noting that this lipid is present in *Desulfobacterota* (62).

### Membrane vesicles.

Gram-negative bacteria form OMVs (40, 41, 65–69). OMVs mediate intercellular communication via the transfer of a wide variety of molecular cargoes (70). They also aid with horizontal gene transfer and microRNA-based reprogramming (71). It has been proposed that mitochondrial OMVs are a defense mechanism for the mitochondria to eject damaged proteins or nucleic acids in order to avoid the failure of this organelle (70). The functions of the morphologically diverse OMVs observed in the environmental *Desulfobacterota* here remain unknown but may be a common feature in the syntrophic bacterial partners of ANME archaea, as they were also observed in the ANME-SRB mats in the Black Sea (72). In the context of the involvement of these syntrophic SRB in direct extracellular electron transfer with ANME archaea (34, 73), it is notable that metal-reducing *Shewanella* produce redox active OMV’s (74) and have recently been shown to engage in extracellular electron transfer via periplasmic extensions of the outer membrane (75). The molecular mechanisms for selecting the cargo to be inserted into the OMVs are not well-understood (76), but a key factor may be the oxidation of the cargo (77). A molecule that may be involved in the budding of OMVs is the bacterial homolog to the protein kinase PINK1 (PTEN induced kinase 1; UniProt identifier: Q9BXM7), which aids in the budding of OMVs from mitochondria (77) and has homologs in many bacterial lineages, including *Desulfobacterota* (Table S1; also cited above). Understanding the molecular mechanisms and ecophysiological factors that trigger the formation of OMVs in the syntrophic *Desulfobacterota* and identifying the cargo that they carry will be important next steps in understanding the interactions occurring within and between syntrophic SRB and ANME archaea cells in consortia.

### Prospectus.

Membrane curvature is generally mediated by specific proteins (78), and our observation of membrane invaginations in *Desulfobacterota* opens an avenue for identifying the proteins involved in this. Alphaproteobacteria that form ICMs appear to have a MICOS equivalent (9). However, no MICOS equivalent (reviewed in references [9] and [21]) has been found yet for *Desulfobacterota*. Because pera and pera junctions resemble lamellar cristae and crista junctions, respectively, a future research target is to discover what molecular machinery is involved with their formation. Crista formation in opisthokonts involves two distinct pathways (79). Tubular cristae are formed independently of Opa1, a protein involved in mitochondrial fusion. In contrast, Opa1 is the anchor protein for the pathway for lamellar crista formation. Because Opa1 is dispensable for crista junction formation (80), its absence in in the cells studied here could explain the observation of a subset of pera not connected to the cytoplasmic membrane (Fig. 5D, H, K). In addition, MICOS proteins, ATP synthase dimers, and cardiolipin are necessary for cristae formation. Because pera have only been observed in the lamellar form, it would be interesting to determine whether *Desulfobacterota* have a functionally equivalent Opa1 homolog. Thus, not only will it be important to identify the proteins involved in pera junction formation and whether cardiolipin aids in the bending dynamics at the junction opening but it will also be useful to determine which energy-conserving proteins reside in the pera and to try to understand whether their dense packing makes energy harvesting more efficient.

What are the possible phenotypic consequences of compartmentalized bioenergetics within the *Desulfobacterota*? In mitochondria, the cristae structure is related to the mitochondrial state (81), and the cristae can differentially separate the membrane potential (82). For the cells observed here, almost nothing is known about the bioenergetic consequences of the ICMs, but it is worthwhile to note the respiratory rate differences between the organisms studied here: *D. carbinolicus* grown in the lab has a doubling time of approximately 3 to 6 h, (83), whereas the syntrophic sulfate-reducing bacteria which partner with ANME archaea double on the order of months (34). Since this difference in doubling time would likely correlate with the sulfate respiratory rate, the co-occurrence of pera in these different cell types with different growth rates is intriguing. A possible line of future research is to link structural observations with modeling efforts, which have opened the door to the linking of cellular energetics, respiratory flux, and stable isotope fractionation (84, 85). These works can help us better understand energy allocation as well as the energetic state of cells in deep time (86), but, so far, models have not considered cellular architecture; compartmentalization could potentially shift intracellular reaction energies, and investigating this is an exciting target for future research, which could involve modeling approaches that could help with the enumeration of the physical parameters that determine membrane shape (87).

## MATERIALS AND METHODS

The site location and sample description of the ANME-SRB consortia recovered from deep sea methane seep sediments are available in the Supplemental Material (Text S1).

10.1128/mbio.01613-22.1 TEXT S1 File containing (1) the site location and the sample description of the ANME SRB consortia as well as (2) proposed future stain work. Download Text S1, DOCX file, 0.03 MB. Copyright © 2022 McGlynn et al. 2022 McGlynn et al. https://creativecommons.org/licenses/by/4.0/ This content is distributed under the terms of the Creative Commons Attribution 4.0 International license.

### Preparation of Desulfovibrio carbinolicus cells.

*D. carbinolicus* was grown in a carbonate buffered medium containing (per L): NaHCO_3_, 5 g; Na_2_SO_4_, 3 g; KH_2_PO_4_, 0.2 g; NaCl, 2.5 g; MgCl_2_·6H_2_O, 1.3 g; CaCl_2_·2H_2_O, 0.15 g; KCl, 0.5 g; resazurin, 1.1 mg; 1 mL of trace element solution SL-10; 10 mL of a vitamin solution described as a part of DSMZ medium 141 (catalogue of strains 1993; DSMZ, Braunschweig, Germany); and 1 mL of tungsten-selenium stock solution (4 mg of Na_2_WO_4_·2H_2_O and 3 mg of Na_2_SeO_3_·5H_2_O per 1 L of 12.5 mM NaOH). Sodium ascorbate (5 mM) and titanium (III) chelated by nitrilotriacetate (NTA; 60 μM) were added as reducing agents, and ethanol (20 mM) was used as an electron donor in all of the incubations, though the electron donor in the inoculum varied as described below. The medium was titrated to a pH of 7.2 and prepared anaerobically under 80% N_2_ and 20% CO_2_ gas. Batch cultures were incubated at room temperature, and growth was monitored by measuring the optical density at 660 nm. Cells were harvested by centrifuging 5 to 10 mL of culture in the early exponential phase (DC3) that were inoculated from a culture grown on ethanol, late exponential phase (DC1) inoculated from a culture grown on methanol, and early stationary phase (DC2) inoculated from a culture grown on methanol, and they were then fixed with 2.5% glutaraldehyde in 50 mM HEPES buffer. The fixed cell suspensions were stored at 4°C until processed.

### Isolation of microbial consortia.

To visualize the cell membranes and structure, we adapted a protocol developed at the National Center for Microscopy and Imaging Research (NCMIR San Diego, CA) that was originally designed for the serial block face scanning electron microscopy (SBEM) of biological specimens (88). For our purposes, this protocol was initiated after the fixation of our samples. Sediments were fixed in an equal volume of sediment to fixative, with the fixative comprised of 4% paraformaldehyde and 5% glutaraldehyde in 18.75 mM HEPES buffer (pH 7.4) containing 13.1 g/L NaCl, thereby yielding a final aldehyde concentration of 2.5% glutaraldehyde and 2% paraformaldehyde, consistent with a protocol described previously (35).

To isolate the microbial consortia, the fixed sediment was washed 4× with 50 mM HEPES (pH 7.0) 35 g/L NaCl by centrifuging for 2 min at 2,000 rcf, removing the supernatant, and then resuspending in 1 mL of the above buffer. After this washing, 750 μL of the slurry was sonicated on ice using a Branson Sonifier W-150 ultrasonic cell disruptor with a sterile remote-tapered microtip probe (Branson) inserted into the liquid. Sonication was carried out in two bursts. The first burst lasted for 30 s, and then, after resting for 15 s on ice, the second sonication was a 15 s burst. Both bursts were at a setting of 3 (approximately 6V [rms] output power). 750 μL of Percoll was then added to the bottom of this tube via pipet, and the resultant mixture was centrifuged at 4°C for 20 min. After centrifugation, the consortia containing the supernatant was removed from the tube. Percoll was removed from this solution via buffer exchange (three separate additions of approximately 10 ml volumes) over a 25 mm, 3 μm TSTP filter that was placed onto a 15 mL glass filter tower that was fitted with a vacuum line. After these three buffer additions were performed to remove the Percoll, the solution was concentrated over the 3 μm filter to an approximate volume of 1 mL or less, and this was removed from the filter tower into a 1,000 μL pipet tip. The filter tower was removed, and, finally, the Percoll material was concentrated by pipetting the solution slowly from the pipet tip onto the 3 μm filter in a small area (~2 mm diameter). Immediately after this, the material was overlaid with molten agar (2% Difco Nobel Agar in 50 mM HEPES [pH 7.4]; 35 g/L NaCl). Once cooled (approximately 20 s), the agar plug containing the concentrated consortia was peeled from the filter, sliced into 1 to 2 mm pieces with a razor blade, and stored in 50 mM HEPES buffer (pH 7.4) and 35 g/L NaCl at 4°C until use (as originally described [34]). The processing for the EMT, data collection, and volume segmentation were performed as described (44, 88). 3,3′-diaminobenzidine (DAB) labeling was applied to the ANME-microbial consortia as described (34).

### Measurements and statistics.

Measurements were made using IMODinfo and ImageJ. The statistics package of Microsoft Excel was used for the statistical comparisons. The mean, standard deviation, standard error of the mean, median, and first and third quartiles were calculated for all reported measurements and are shown in box-and-whisker plots that were created using BoxPlotR (http://shiny.chemgrid.org/boxplotr/). The individual data points are shown in the “bee-swarm” configuration. The whiskers (vertical lines) extend from the ends of the box to the minimum value (bottom) or the maximum value (top). A data point was considered to be an outlier if it exceeded 1.5 times the interquartile range below the first quartile or above the third quartile. An analysis of variance (ANOVA) was used with Bonferroni’s *post hoc* correction, using the two-tailed unpaired Student’s *t* test between groups. *P* values that were <0.05 were considered to be indicative of a statistically significant result. When the stricter criterion of *P* < 0.01 was reached, it was reported instead.

### Data availability.

All raw data and materials that are relevant to the publication will be freely available to any researcher wishing to use them for noncommercial purposes, respecting participant confidentiality.
