# Supplementary material for: A Cristae-Like Microcompartment in Desulfobacterota
Source: mBio. 2022 Nov 2;13(6):e01613-22. doi: 10.1128/mbio.01613-22 (PMC9764997; doi:10.1128/mbio.01613-22)
Supplement: TABLE S1 [file mbio.01613-22-s0004.docx]

**Table S1.** Bacterial Homologs of Known Proteins Involved in Cristae Formation

| **Protein** | **PFAM** | **Result from searching with Annotree (PFAM search with e-value of 0.00001, AnnoTree v1.2.0; GTDB Bacteria Release R95; GTDB Archaea Release R95; Pfam v27.0)** |
| --- | --- | --- |
| Vps35 | PF03635 | Two genome hits in bacteria at e-value of 0.00001. These    are in Firmicutues. |
| RAB7A (Mic1) | PF00071 | Widespread in bacteria, present in Alphaproteobacteria and Desulfobacterota. |
| Parkin | PF01485 | Present in 24 bacteria, including 2 Desulfobacterota (sp002840535 and sp002419385) |
| Opa1 | PF00350 | Widely spread in bacteria including Desulfobacterota. |
| PINK1 | PF00069 | Widespread in bacteria |
| Mic10 | PF04418 | Found in 1 Desulfobacterota (UBA5852 sp002432195), 1 Firmicutes A, and 1 Proteobacteria using Annotree. |
| Mic12 (Qil1) | PF17050 | Not present in Annotree as a query. |
| Mic60 | PF09731 | Not found in Desulfobacterota using Annotree. In Alphaproteobacteria. |
| Mic26 | PF09769 | Present in 15 bacterial genomes: 2 Actinobacteriota, 1 Bacteroidota, 2 Desulfbacterota (Desulfovermiculus halophilus and Humidesulfovibrio sp002840405), 1 FCPU426, 8 proteobacteria, and 1 Verrucomicrobiota |
| Mic19 | PF05300 | Present in 1 Firmicutes |
