# Supplementary figures and images for: A Cristae-Like Microcompartment in Desulfobacterota
Source: mBio. 2022 Nov 2;13(6):e01613-22. doi: 10.1128/mbio.01613-22 (PMC9764997; doi:10.1128/mbio.01613-22)

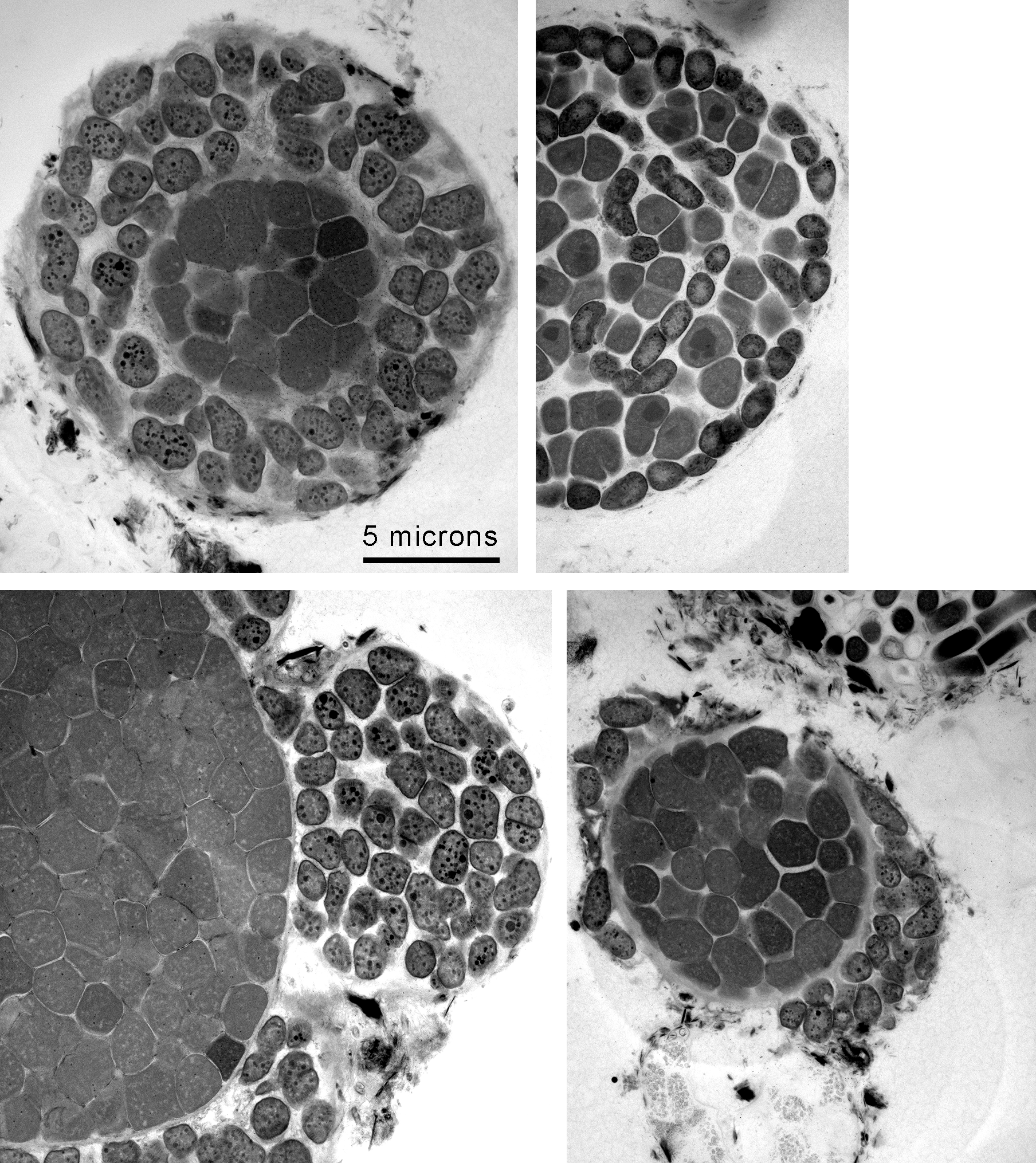

Supplement: FIG S1 [file mbio.01613-22-s0002.tif]

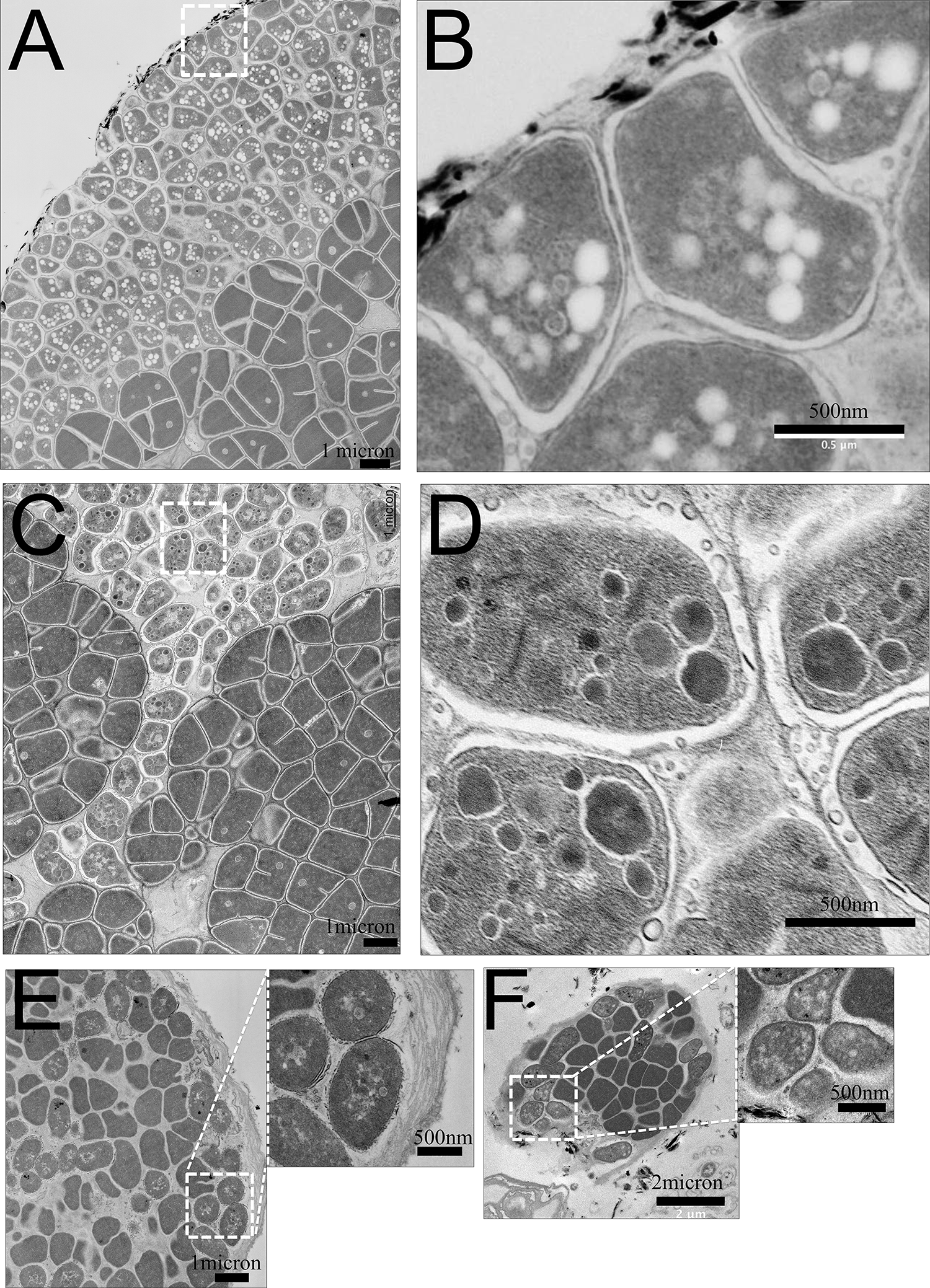

Supplement: FIG S2 [file mbio.01613-22-s0003.tif]
