# Supplementary material for: A Cristae-Like Microcompartment in Desulfobacterota
Source: mBio. 2022 Nov 2;13(6):e01613-22. doi: 10.1128/mbio.01613-22 (PMC9764997; doi:10.1128/mbio.01613-22)
Supplement: TEXT S1 [file mbio.01613-22-s0001.docx]

**Supplementary Information**

1. **Site location and sample description of ANME SRB consortia**

The microbial communities associated with the 2 seep sediment incubations were previously described; 5133 (1); 3730 (2)(3) and see also (4)(5). Here, bacteria paired with archaea were structurally analyzed, and although current protocols involving staining for EMT preclude paired TEM-FISH, these bacteria are likely to be *Desulfobacterota* partners: A fluorescence *in-situ* hybridization survey of the 5133 sediment incubation showed that archaea-bacteria containing multicellular aggregates were 27% archaea with *Desulfobacterota* targeted by the delta495 probe + competitor probe (6)(7), and 26% archaea paired with *Desulfobacterota* targeted by the seep1a-1441 probe (8). 6% of the consortia were archaea paired with an unidentified microbial partner, and the remainder of the aggregates were comprised of only archaeal cells, or clusters of *Desulfobacterota*/Seep1a, unassigned consortia not targeted by the archaeal probe (likely bacterial aggregates), or ANME-1 with and without a bacterial partner.

Throughout this manuscript, seep sediment incubation 5133 is referred to as T2B, and incubation 3730 as T3B. These designations were made for reference and archival purposes.

Seep sediment microcosm incubation 5133: The 5133 sample was collected during the R/V Atlantis expedition AT 18-10 in August and September of 2011 using the ROV Jason II. Sediment sample 5133 originated from the upper 9 cm depth horizon of a push core (30 cm long) collected within a white microbial mat from an active methane seep located at Hydrate Ridge North (station HR-7; 5294, 13003; 600 m water depth). The push core was processed shipboard immediately following recovery with the mat removed and the upper 9 cm of sediment extruded from the core and transferred into N_2_ flushed mylar bag. This sample was heat sealed and stored at 4º C until transport back to the lab. In the shore based lab, sediments were kept cool on ice and transferred into a sterile 1L Pyrex bottle in an anaerobic chamber, mixed with chilled N_2_ sparged 0.22 µm filtered bottom water from Hydrate Ridge at a ratio of 1:3 sediment to liquid, and sealed with a large butyl stopper. The sediment incubation was then over pressurized with a CH_4_ headspace (30 psi) and incubated in the dark at 6º C. This large sediment microcosm maintained active sulfate-coupled methane oxidation in the laboratory and was sampled periodically to obtain fresh sediment for the investigations described here. Sediment incubation “3730” was collected in 2010 during cruise AT15-68 on dive AD4635 using the human operated vehicle Alvin. This sample represented the top 0-6 cm sediment collected using a push coring device within a Calyptogena clam bed exhibiting active methane bubbling (Hydrate Ridge South, 44 44º 34.09N; 125º 9.14; 775m water depth). Sediments were immediately transferred into a 1 L Pyrex bottle, flushed with N_2_ gas, stoppered and maintained at 4º C. A sediment slurry (500 mL) was subsequently created by mixing one volume of the collected sediment with two volumes 0.22 µm filtered bottom water from Hydrate Ridge in a Coy anaerobic chamber. Samples were over pressured with methane and incubated as described above.

1. **Proposed future stain work**.

In our previous work, we developed methods for pairing fluorescence *in-situ* hybridization (FISH) observations with those made on TEM (6). In that study, the chemical staining needed to gain high TEM contrast was not used, since it disrupts FISH signals. A next step will be to apply stains and probes in a manner which allows a view of fine structure and phylogenetic affiliation. In this way, the uncultured majority might remain uncultured, but not unobserved.

**References**

1. McGlynn SE, Chadwick GL, Kempes CP, Orphan VJ. 2015. Single cell activity reveals direct electron transfer in methanotrophic consortia. Nature 526:531–535.

2. Hatzenpichler R, Connon SA, Goudeau D, Malmstrom RR, Woyke T, Orphan VJ. 2016. Visualizing in situ translational activity for identifying and sorting slow-growing archaeal-bacterial consortia. Proc Natl Acad Sci U S A 113:E4069-4078.

3. Trembath-Reichert E, Green-Saxena A, Orphan VJ. 2013. Whole cell immunomagnetic enrichment of environmental microbial consortia using rRNA-targeted Mag-FISH. Methods Enzymol 531:21–44.

4. Martin W, Hoffmeister M, Rotte C, Henze K. 2001. An overview of endosymbiotic models for the origins of eukaryotes, their ATP-producing organelles (mitochondria and hydrogenosomes), and their  heterotrophic lifestyle. Biol Chem 382:1521–1539.

5. McGlynn SE, Chadwick GL, O’Neill A, Mackey M, Thor A, Deerinck TJ, Ellisman MH, Orphan VJ. 2018. Subgroup Characteristics of Marine Methane-Oxidizing ANME-2 Archaea and Their Syntrophic Partners as Revealed by Integrated Multimodal Analytical Microscopy. Appl Environ Microbiol 84.3.

6. Macalady JL, Lyon EH, Koffman B, Albertson LK, Meyer K, Galdenzi S, Mariani S. 2006. Dominant microbial populations in limestone-corroding stream biofilms, Frasassi cave system, Italy. Appl Environ Microbiol 72:5596–5609.7.

7. Loy A, Lehner A, Lee N, Adamczyk J, Meier H, Ernst J, Schleifer K-H, Wagner M. 2002. Oligonucleotide microarray for 16S rRNA gene-based detection of all recognized lineages of sulfate-reducing prokaryotes in the environment. Appl Environ Microbiol 68:5064–5081.1.

8. Schreiber L, Holler T, Knittel K, Meyerdierks A, Amann R. 2010. Identification of the dominant sulfate-reducing bacterial partner of anaerobic methanotrophs of the ANME-2 clade. Environ Microbiol 12:2327–2340.
